# Migraine1Presidents address before the Medical Society of the County of Cattaraugus at Salamanca, N. Y., May 1. 1906.

**Published:** 1906-07

**Authors:** Clarence King

**Affiliations:** Machias, N. Y.


### BUFFALO MEDICAL JOURNAL.

VOL. LXI. JULY, 1906. No. 12

INDEX THROUGH

### ORIGINAL COMMUNICATIONS.

### Migraine.[1]

BY CLARENCE KING. M. D., Machias, N. Y.

THE term migraine properly refers to a severe pain limited to one lateral half of the head and generally accompanied by nausea or vomiting, and by other functional and indefinite symptoms of a character nearly constant to the individual. But while such cases are quite frequent and cause much suffering and inconvenience from their severity and usually obstinate and recurring nature, still there are many milder and less typical cases in which the character of the pain and the disease as a whole is much different. By common consent the term is now applied to any pain of the head which is attended by nausea or vague paresthesias or other symptoms and which is not due to rheumatism of the scalp, to supra-orbital or other neuralgia or to obvious organic lesion.

The causes of migraine are not well understood. Probably the one of first importance is heredity and Gray asserts that this is more often operative through the maternal than through the paternal ancestry. On the other hand the influence of heredity is stoutly denied by many competent observers. Some authors have contended that there is a close relation between this disease and petit mal or other forms or nervous irritability and cite its frequent occurrence in families thus affected. Toxemia, either through the manufacture and absorption of new substances in the alimentary canal or through failure of excretion of some of the poisonous end-products of destructive metabolism, is undoubtedly a potent cause in many instances. Thus, following a debauch or the ingestion of indigestible substances, we often find a headache which though similar in character to that of migraine is relieved by a brisk cathartic or free emesis. But again, stomach digestion seems to be but little interfered with in this disease which lends favor to the opinion that the fault is with the liver or other

1. Presidents Address before the Medical Society of the County of Cattaraugus at Salamanca, N. Y., May 1. 1906.

organs of digestion. Sedentary habits, the failure of proper ventilation, neurasthenia, the menstrual epoch and overindulgence in the pleasures of the table or in venery are not without influence in those predisposed. But while these causes, as likewise anemia, insomnia, arteriosclerosis and eye strain, are undoubted factors in causing ordinary headache it is very doubtful if they alone are ever able to induce true migraine.

Sick headache occurs more often in women than in men, possibly in part due to their more sedentary employment which tends to make excretions less active and affording greater opportunity for toxic absorption. It is a disease, essentially, of early adult life, rarely beginning before puberty or continuing to very advanced age. When once initiated attacks of more or less severity are apt to recur for a long period of years either with regularity or upon exposure to some definite cause; but after middle life they tend to diminish in severity and in frequency, if not totally to cease. This, however, does not invariably happen and numerous exceptions to this rule occur.

A typical attack of migraine is often preceded by premonitory symptoms, of long or short duration, which are pathonomonic to each case, the sufferer himself from past experience or his friends being able to predict an attack some time in advance. These symptoms consist of dimness of vision, of paresthesias of the skin, generally of one or both upper extremities or of the face, of slight nausea and loss of appetite, of chilliness, and of a general feeling of malaise or lassitude. The premonitory stage is followed by an uneasiness in one side of the head, usually over the eyebrows or in the temples, which soon merges into actual pain, sometimes very intense in character. With this pain there may be intolerance to light and sound and the patient lies quietly with the eyes closed or he may be drowsy and sleeps. In other cases the pain may be slight and the premonitory symptoms wanting or at least transient and not well marked; but in all cases there is more or less nausea at some time during the attack. Frequently the emptying of the stomach by emesis gives partial relief for a time, especially if the attack is induced by overindulgence, or it may terminate it if it has lasted for some hours. But again this has no appreciable effect on the sickness, the only relief coming from opiates or analgesiacs or from the attack gradually wearing itself out.

The severity of an attack of migraine varies between wide limits and is not always proportional to the actual amount of pain present. Thus, a person with little pain may vomit more, suffer more from paresthesias of various parts, from photophobia and dimness of vision and be as much incapacitated from business as one who has more pain. Again, the cases in which pain is not intense but in which the other symptoms are severe show as much tendency to recurrence at regular or irregular intervals as the very painful ones, at least during the time of greatest activity of the disease. As regards danger to life it may be said that migraine is never followed by fatal results; nor does it often, if ever, induce other diseases. But that it tends to recur with more or less regularity for a long time and to cause absolute suspension of the usual business activity of the sufferer is a matter of common observation with us all.

As an interesting, although quite typical, example I would cite the case of a former medical friend in Buffalo in whose home I was an inmate during two winters while taking my college course in medicine. This gentleman had been a sufferer from migraine for years, having inherited it from his maternal ancestors. Once a month, with almost the regularity of a woman's menses, he had an attack which lasted three days, the two last days being very severe. On the first day he was able to be about and attend to business, making outside calls to those he ought to see during his attack and telling them he could not visit them again until it was past. The second and third days he remained in bed, no one but his immediate family seeing him until the evening of the third day when he would come down to lunch, scarcely able to drag himself to the dining room and looking as though he had passed through a prolonged and severe sickness. The following day he would be in his usual health again. Up to that time he had been unable to find anything that helped him in the least but some years afterward he told me antipyrin relieved him somewhat, but that he had become frightened and would not use it on account of the toxic effects he had experienced at one time after taking it.

The treatment of migraine is, on the whole, very unsatisfactory, as more or less severe attacks are apt to recur in spite of any treatment yet devised and only cease with the advent of advanced age or possibly in exceptional cases with a radical change in the personal habits and mode of living. However, the individual attacks may be cut short or made less severe by the use of opiates or some of the coal tar derivatives. We are all familiar with the migraine tablet containing acetanilid, monobromated camphor and citrate of caffeine which in mild cases will frequently bring relief but antipyrin will often succeed where this fails. In severe cases morphia or opium in some form is undoubtedly the safest, if not the only, drug of real use, but it should only be given at intervals and after careful consideration on account of the great danger of inducing the drug habit. Bromide of potassium or soda and chloral hydrate have been employed by some physicians to relieve the attack but I have never seen much benefit follow their use in this disease, while from their irritating effects upon the gastric mucous membrane they are apt to increase the distress at the stomach if not add to the vomiting. This objection, however, may be obviated by using them per rectum.

The treatment of the disease between the attacks offers some chance of lessening their frequency and severity to a moderate degree and for this purpose a large number of drugs have been tried, which goes to show that none of them is entirely satisfactory. The one that has given me the best results is cannabis indica; this I give in doses of one-quarter to one-half grain three or four times a day, generally in tablet form on account of its greater convenience to the patient as it needs to be continued for months or even years. Patients who will persist in this treatment seldom fail to receive benefit even in the worse cases, as the attacks gradually become less severe and the intervals longer; but a return to their former severity follows its discontinuance. I have a patient who has taken the drug for nearly three years with only short intermissions, these being followed by a severe attack of migraine, which caused her to again begin its use. She now asserts her intention of continuing it until advanced age relieves her of its necessity.

In some cases the sodium salts, particularly the benzoate, the salicylate or the sulphate, give relief but in their use I have had little or no experience. The bromides cannot, or at least, should not, be used continuously for the purpose of warding off an attack as the resulting mental impairment is worse for the patient than the occasional attack of migraine. In all cases an attempt should be made to ascertain the cause of the disease and if it is possible to find any accompanying disorder it should receive attention. Thus, we often note these patients are anemic and tire easily, possibly have a blowing murmur at the base of the heart, which is easily excited to increased activity. In such cases a course of iron or arsenic with some bitter tonic like calisaya or quinine, is a good adjunct to other treatment and should not be omitted. Other cases showing lithemia or the gouty diathesis, as indicated by evidences of imperfect oxidation, by catarrhal disturbances of the gastrointestinal tract, by deficient biliary secretion with foul breath and coated tongue, by arteriosclerosis or by chronic diseases of the skin, call for appropriate remedies directed to the accompanying condition whether we accept that as causative or not.

These cases occur most frequently in hearty eaters, especially if leading a sedentary or in-door life and show an excess of uric acid or urates in the urine or a deposit of oxylates or lithic acid crystals upon cooling. A brisk chologogue cathartic followed by a prolonged course of treatment by alkalies with restricted diet and abundance of out-door exercise, will usually improve the general condition and frequently ameliorate the migraine.

Again, if we find astygmatism or disease in the aureal or nasal cavities or in the throat, or if pelvic or cardiac disease is found by careful inquiry or examination, this should be attended to by competent specialists, if necessary, as we can scarcely hope to improve the migraine so long as there is reflex irritation from these parts.

Of late years physical therapy has come largely into favor in the treatment of disturbed metabolism and lowered enervation and migraine offers a promising field for its use. The high potential electric current is a powerful tonic to the whole system and the static breeze and insulation are great equalizers of function. In migraine, as in other forms of chronic disease, they will accomplish much if their use is persevered in for a sufficient length of time. Snow has great faith in the wave current and directs it to be applied to any part most agreeable to the patient but prefers the spine. Often during an attack, if very severe, the galvanic current will be found of use, one electrode being applied to the head; a mild current only can be used, the polarity being determined by the conditions present. Generally the positive pole is indicated on account of the congested state of the brain but the opposite may be called for in some instances.

Again, hydrotherapy, as by the dripping-sheet bath, may be of use in stimulating the nerve centers and arousing normal functionating of the organs. Or, if a history of toxemia can be elicited, the superheated air or steam bath may be employed with good prospects of beneficial results, especially if it can be shown there is deficient excretion or sluggish portal activity; and finally, if constipation is present massage, vibration or electricity in some form, or other physical agent may be of use in causing normal evacuations and bringing about amelioration of the migraine.

I desire now to describe to you the disease as it affects the author of this paper, as it presents some symptoms I have never met elsewhere or seen described in literature. I make no claim to their being unique or unheard of before but they must be at least infrequent and may be somewhat interesting to you.

My maternal grandmother was a sufferer from migraine for many years up to within ten or twelve years of her death at the age of seventy. Within my recollection her attacks were infrequent but lasted several days and were attended with a great deal of dizziness and nausea which confined her to bed. My mother died at age of thirty when I was only a year and a half old but I

have heard my father speak of her as suffering from migraine. My migraine came on when I was a boy in school, probably about ten or twelve, and I had frequent attacks until about twenty-five years ago since which time I have been gradually getting better of them. For a number of years past I have seldom had an attack and when I have, it has not been severe and has lasted only an hour or two. But it is the attack itself, as it affected me at its worst, I wish particularly to describe.

Ordinarily, my family can tell about half an hour in advance that I am going to have an attack of migraine by the appearance of my eyes, but otherwise I have no premonitory symptoms. The attack is ushered in suddenly by two symptoms, either one of which may precede the other by a few minutes. These symptoms are a peculiar disturbance of sight and a paresthesia of the skin, generally of one or both hands and often extending up the arm half way to the elbow. This paresthesia consists of fine prickling sensations which are popularly known as the part being asleep, last only a short time, perhaps two to ten minutes and pass away suddenly. They may, however, recur. Years ago I experienced these sensations in the face when they affected the upper lip principally but sometimes also the tongue. When thus located I always found they betokened a severe attack. When the tongue was the site of these sensations it felt thick and swollen and speech was interfered with, the articulation of words being indistinct or impossible; in other words, a temporary motor aphasia was present

With this paresthesia of the skin there is a disturbance of the eyesight which is peculiar in that the range of vision is limited to a very small area. Thus, if I happen to be looking at a person at the time, I can see his face distinctly but his body and limbs and surrounding objects are entirely gone until I move my eyes to direct them squarely upon these parts. This gives rise to queer preception of objects, as you can readily see, for the head appears as if floating in the air and talking unconcernedly without a body. If I move my eyes downward the body suddenly jumps into view while the head vanishes; in either case there are no limbs.

But this eccentricity of sight is still more characteristic if I happen to be reading at the time. In that case I can see distinctly the one word my eyes rest upon, but above, below and at each side is blank paper to the extent of about four inches when that too is lost. As I move my eyes along the line another word suddenly comes into view while the former one just as suddenly vanishes from sight and it makes no difference how long or how short that word may be. A hyphen between two words gives me a clear perception of them both, while without it I can see but one. The dot over the letter "i" shows in its proper place but if there occurs a little longer break, as between the words or between the lines, my vision can not span it to get even a shadowy idea of what is beyond. This disturbance of vision, as you can see, is not hemianopsia in which one-half the field of vision is lost, nor micropsia in which objects appear smaller than they really are. I have been unable to find a term that expresses the condition and will not attempt to coin one.

With these symptoms I generally have some nausea and occasionally vomit, have chilly sensations and wish to get near a fire, although that may make my general sickness worse. But with all this I never have much, if any, headache; in fact, I have had many attacks with absolutely no pain. I always feel hungry and as soon as nausea subsides can eat a hearty meal. As for treatment I generally take a migraine tablet and a laxative when my distress is great enough to call for anything and drink a cup of hot tea. When my attacks were severe and before we had acetanilid they always lasted me the balance of the day without regard to the time of the day they came on. If I slept two or three hours during the day I felt just as badly upon awaking as I did before going to sleep; nothng would relieve me but a full night's sleep and that invariably would. In closing, let me say that I do not consider my gradual improvement during the last score of years with nearly entire freedom for the last eight or ten to be due to old age, but rather to the active life I have lead during those years, a great part of which has been out of doors, made necessary by the practice of my profession.

